# Why sampling ratio matters: Logistic regression and studies of habitat use

**DOI:** 10.1371/journal.pone.0200742

**Published:** 2018-07-23

**Authors:** Ladislav Nad’o, Peter Kaňuch

**Affiliations:** 1 Institute of Forest Ecology, Slovak Academy of Sciences, Zvolen, Slovakia; 2 Institute of Biology and Ecology, Faculty of Science, P. J. Šafárik University in Košice, Košice, Slovakia; Universita degli Studi di Napoli Federico II, ITALY

## Abstract

Logistic regression (LR) models are among the most frequently used statistical tools in ecology. With LR one can infer if a species’ habitat use is related to environmental factors and estimate the probability of species occurrence based on the values of these factors. However, studies often use inadequate sampling with regards to the arbitrarily chosen ratio between occupied and unoccupied (or available) locations, and this has a profound effect on the inference and predictive power of LR models. To demonstrate the effect of various sampling strategies/efforts on the quality of LR models, we used a unique census dataset containing all the used roosting cavities of the tree-dwelling bat *Nyctalus leisleri* and all cavities where the species was absent. We compared models constructed from randomly selected data subsets with varying ratios of occupied and unoccupied cavities (1:1, 1:5, 1:10) with a full dataset model (ratio 1:31). These comparisons revealed that the power of LR models was low when the sampling did not reflect the population ratio of occupied and unoccupied cavities. The use of weights improved the subsampled models. Thus, this study warns against inadequate data sampling and highly encourages a randomized sampling procedure to estimate the true ratio of occupied:unoccupied locations, which can then be used to optimize a manageable sampling effort and apply weights to improve the LR model. Such an approach may provide robust and reliable models suitable for both inference and prediction.

## Introduction

Logistic regression (LR) models or their extensions are among the most frequently used statistical tools in ecology for analysing the distribution or habitat selection of species [1]. With LR one can infer if habitat use is related to environmental factors and estimate the probability of species occurrence based on the values of these factors [2]. Both outputs—inference and prediction—are foundations for developing effective evidence-based nature conservation policies and management [3].

In their influential study [4] Keating & Cherry reviewed frequently used sampling designs and how they influenced the reliability of obtained models. They recommended that LR should be used only in habitat studies with random sampling, when one large sample of locations is randomly chosen from an environment and then afterwards examined and classified into two groups (occupied and unoccupied). Such a data collection strategy insures unbiased assessment of the ratio between occupied and unoccupied locations and provides a better estimate of the population ratio (i.e. the underlying ratio between occupied and unoccupied locations in environment). Models constructed from data so collected can be used directly to estimate the probability of habitat use.

In comparison, LR models derived from a case—control design, which yields two samples—one of occupied and other of unoccupied locations—should be used with caution. This is because the relative proportion of occupied and unoccupied locations is determined by the researcher, and therefore it may not be representative of the underlying population ratio. These models are not suitable for estimating the probability of occurrence and must be evaluated only in terms of odds ratios. Thus, they are only useful for estimating how often a location is likely to be (un)occupied in comparison with another location. Estimating the probability of occurrence under a case-control design is possible only if the underlying population ratio is known; appropriate weights are then applied to occupied and unoccupied locations during creation of the LR model.

Both of these samplings strategies—randomized and case—control—should only be applied in situations when the researcher can unambiguously distinguish between occupied and unoccupied locations, for example, in the case of a conspicuous, less mobile animal species that is easy to detect. However, LR is also very frequently employed in studies where the absence of a species in the resource units of an environment is uncertain. This is basically the case of highly mobile and hard-to-detect animal species. In such cases, researchers are only able to identify occupied locations with high accuracy (by radio- or GPS-tracking techniques), but they do not know whether some locations were truly not used by un-tracked individuals (due to tracking or observing only a limited set of individuals). The problem of uncertain determination of locations is often solved practically by treating these studies as equivalent to a case—control design, under the assumption that the probability of animal presence in the control sample is very small [4].

Unfortunately, more than a decade after publication of a review that warned against misinterpretation of LR [4], models are often employed without special caution, especially by ignoring the problem of arbitrarily chosen ratios of occupied and unoccupied (or available) locations. Correct sampling in use—availability design is essential if a logistic model is to be used for inference or probability prediction of habitat use [5]. In this paper, we first briefly review several characteristics of recently published studies that use LR to detect habitat use or selection. We focus particularly on the most frequently used sampling designs (i.e. the population ratio of occupied and unoccupied locations). We then use the tree-roosting Leisler’s bat, *Nyctalus leisleri*, to demonstrate the effects of various sampling ratios of occupied and unoccupied locations (1:1, 1:5, 1:10 and underlying population ratio) on the parameters of the obtained LR models depicting the roost use. To maintain simplicity in this demonstration, we employed only one habitat variable to infer potential selection of bats and to estimate the probability of bat occurrence: the height of the roosting cavity. Thanks to the properties of our unique dataset, which contained the heights of all cavities in the study population home range, and long-term radio-tracking of individuals, we constructed a full model from all occupied and unoccupied cavities.

We compared models from variously subsampled data sets with the full model to test the effects of different sampling ratios on model robustness. We also studied whether the robustness of model inference and prediction can be improved by applying weights to data points. Finally, in this article we discuss a sampling procedure for estimating the population ratio of occupied and unoccupied locations, which can be used to optimize a manageable sampling effort and to apply appropriate weights to improve the LR model.

## Materials and methods

### Literature overview

A literature search using the Web of Science^™^ (All Databases) database for peer-reviewed papers published from 2005 to 2016 (the combination of topic keywords ‘(logistic regression* ecology* habitat selection*) AND (telemetry OR tracking)’) resulted in a list of 59 papers (S1 List) in ecological and/or zoological journals (a full list of the web search resulted in 100 papers; however, 41 papers were not considered, as they did not report the sampling ratio between occupied and unoccupied/available locations or were irrelevant to our purpose). The studies were examined for the sampling design employed, and findings were visualized using pie plots.

### Study species and data collection

Leisler’s bat (*Nyctalus leisleri*) is a medium-sized, insectivorous vespertilionid species inhabiting a predominantly forested landscape in temperate zone throughout most of Europe. It roosts almost exclusively in tree cavities [6] and, during the breeding season, maternity groups of adult females and their young switch these roosts every several days [7].

We collected data in the Pliešovská Kotlina Basin (Gavurky Protected Area, central Slovakia; N48°27’, E19°08’; 470 m a.s.l.) in an old pastured woodland habitat of 81.8 ha, characterized by oaks (*Quercus robur* group, *Q*. *cerris*) ranging in age from 200 to 300 years old. Oaks (n = 824 trees) with a median diameter at breast height of 1.1 m (max 2.0 m) provide plenty of roosting opportunities for tree-dwelling bats (n = 932 tree cavities). Over multiple seasons we measured several parameters for each tree (GPS coordinates, DBH, height, number of cavities, bark loss) and for each cavity (height, position, origin). Heights of trees and cavities were measured using a clinometer (Suunto PM-5/360PC)

**Fig 1:**
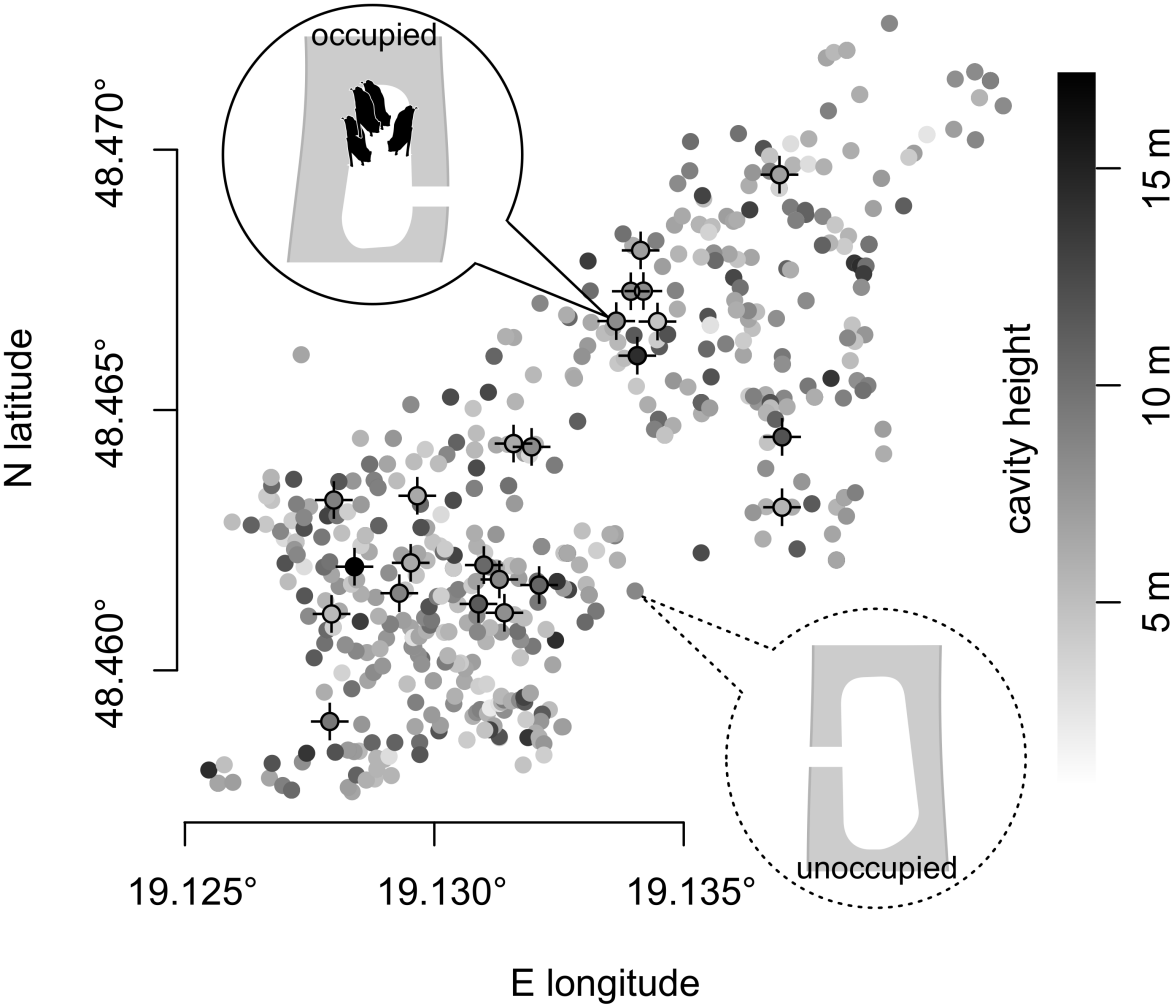
Locations of tree cavities in the study area. Their entrance height is denoted by the shade of the grey fill point (cavities in the same tree are overlapping). The population ratio between occupied and unoccupied cavities is 1:31.

Cavities used for roosting were found by radio-tracking 19 adult females equipped with Pip3 radio-transmitters (Biotrack, Ltd.) using a TRX-3S receiver and a three-element hand-held directional Yagi antenna (Wildlife Materials, Inc.). Bat females form relatively large groups that occupy a single roost. Therefore, tracking only one individual at a time is usually sufficient to keep track of the whole group (several times we tracked more than one individual simultaneously, but the tracked individuals always roosted together). During 148 days of tracking bats in the area (on average 37±14 days per season) we found 29 tree roosts (hereafter also referred to as occupied locations) that were occupied by maternity groups in 2011–2014 (Fig 1). Over a four-year period, bats repeatedly occupied only those 29 roosts. These occupied cavities represented only a very small proportion (~3.5%) of the all tree cavities available in the study area. Therefore, we assumed that none of the unoccupied cavities in our sample was used by bats. This enables us to precisely estimate the underlying population ratio between occupied and unoccupied cavities. The capture and marking of bats were performed as quickly as possible and approved by the Ministry of the Environment of the Slovak Republic (permission Nos 2598/715/03-5.1/pil and 5376/2009-2.1/jam).

### Statistical analysis

Since many textbooks on statistics cover the fundamentals and mathematical details of LR (e.g. [8]), we decided to omit all mathematical discussions herein and instead focus on basic principles explained in simple terms that are clearly comprehensible even to a field ecologist with a limited understanding of technical jargon. In order to mimic scenarios with various sampling efforts, we made one hundred LR models (function “glm” with parameter “family = binomial”), hereafter referred to as subsampled models, for each sampling ratio (i.e. 1:1, 1:5, 1:10). These subsample models were subsequently tested by “anova” function with the parameter “test = Chi”. The data from which these models were constructed consisted of the height of the 29 occupied cavities and 29, 145 and 290 heights of randomly chosen unoccupied cavities (300 models were constructed in total). Each model was computed with the same data set of occupied locations and a different data set of unoccupied locations (S1 Table). The aim of the analysis was to mimic the case—control sampling strategy with an arbitrarily varying number of unoccupied locations. Furthermore, one LR model was constructed from the height of the 29 occupied cavities and the height of all 903 unoccupied cavities (i.e. a ratio of approximately 1:31; hereafter the full model).

There are two reasons why we chose the height of the cavity as the single variable to predict the probability of bat presence. First, we wished to keep our demonstration as simple as possible. Second, the predominant use of cavities with higher entrances has already been found in a different population of Leisler’s bat [9]. Subsequently, we performed a comparison of the parameters (β_0_, β_1_) and significance of cavity height (P-value) between the subsampled and the full model. All subsampled models were than recalculated with weights that were added to the data points from unoccupied localities to approximate the underlying population ratio between occupied and unoccupied cavities. Weights for the unoccupied cavities in the models were thus as follows: sampling 1:1 = 31, 1:5 = 6.2, 1:10 = 3.1. The variability of the fitted values of non-weighted and weighted models was visualized in plots using logistic curves and the variability of their estimated coefficients (mean ± SD) using histograms. All statistical computations were performed using the R 3.2.3 environment for statistical computing [10].

## Results

### Literature overview

**Fig 2:**
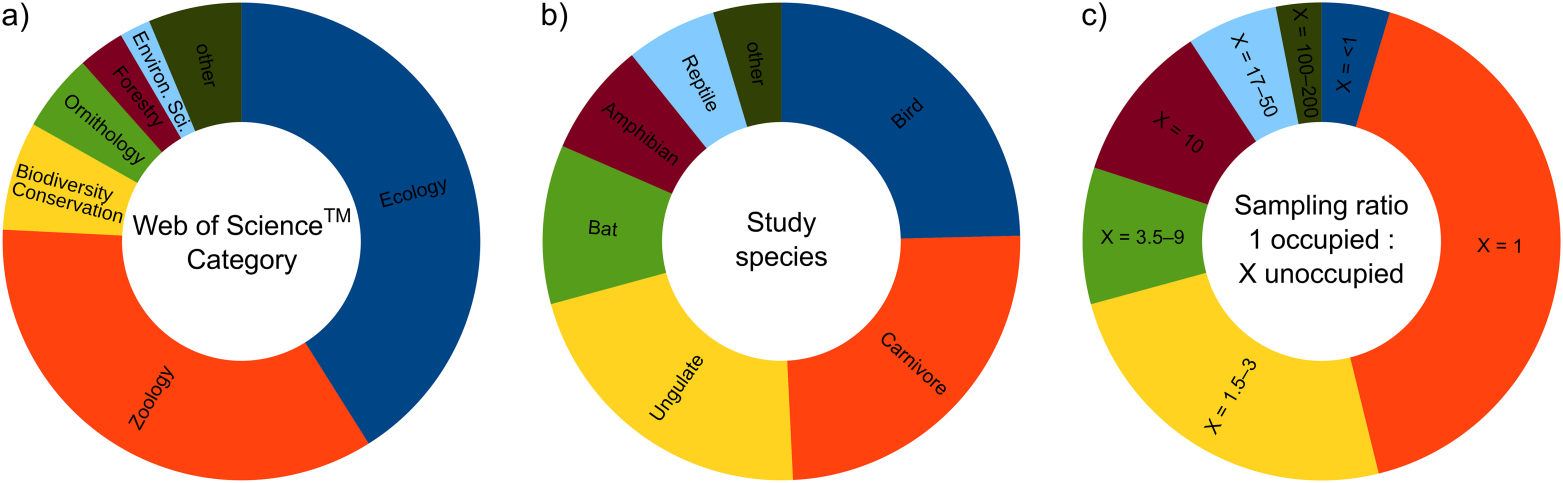
Characteristics of habitat selection studies published in peer-reviewed journals (n = 65 studies in 59 papers) indexed in the Web of Science^™^ (All Databases) database in the time span 2005–2016 according to a) subject category of the journal, b) taxonomic classification of the studied species and c) sampling ratio between occupied and unoccupied (available or pseudo-absent) locations.

Altogether, the examined papers reported habitat selection in 65 samples based on use—availability data which had various sampling ratios, predominantly 1:1 or up to 1:3, and these ratios were chosen arbitrarily without any reasoning. Moreover, these studies modelled habitat selection using tracking data collected in only a few selected individuals that represented only a fraction of the total population, mostly in large mammals, birds or bats in which high mobility, large home-ranges or small body size or even nocturnal activity increased the uncertainty in determining the unoccupied (i.e. available) samples in the published models (Fig 2).

**Fig 3:**
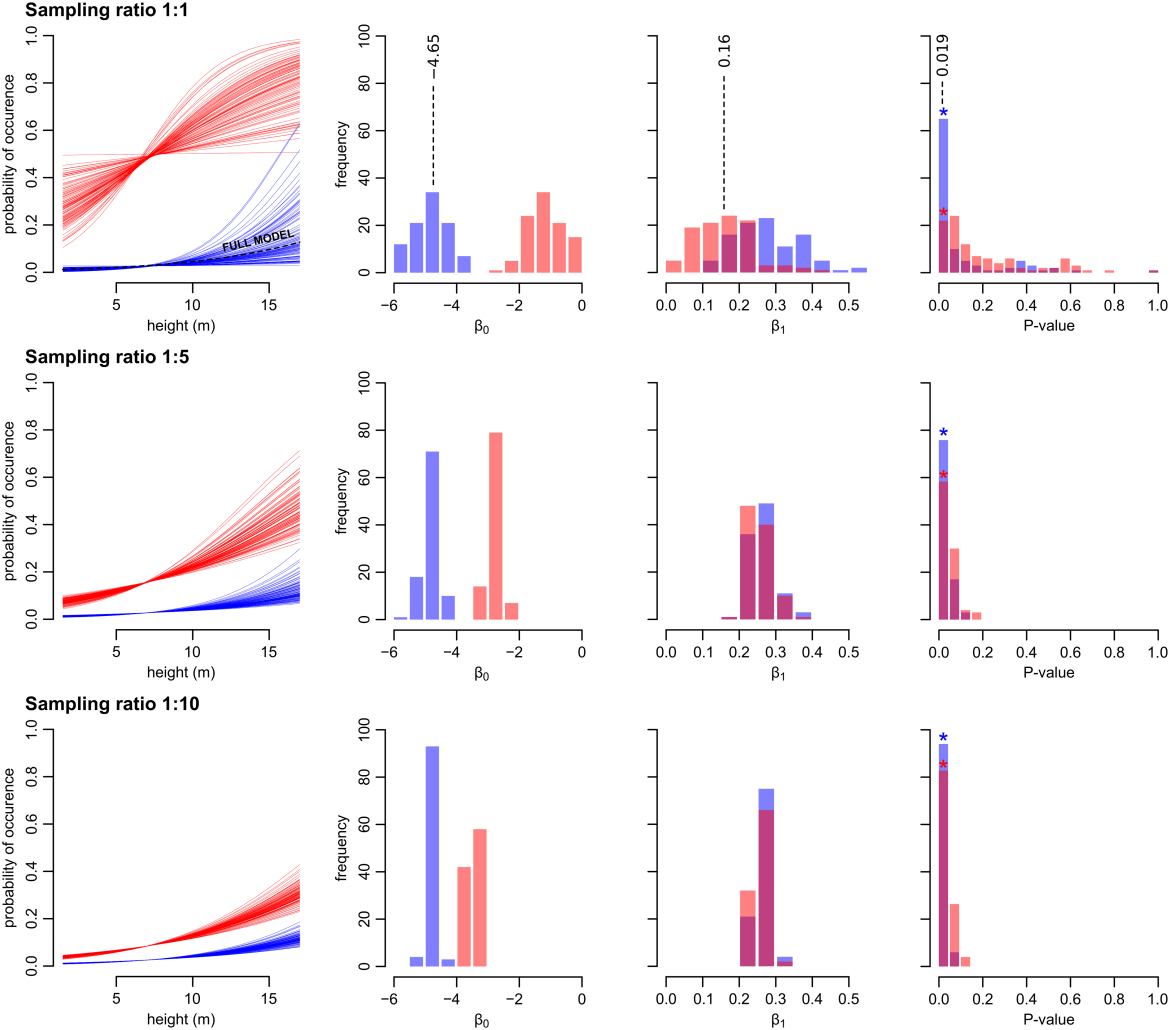
Logistic regression curves, coefficients (on logistic scale) and P-values of models constructed from the set of occupied locations (n = 29) and 100 randomly subsampled subsets of unoccupied locations in three different sampling ratios (non-weighted models in red, weighted in blue). The coefficient values and P-value of the full model are denoted by dashed lines.

The estimates of coefficients and the predicted occupancy probabilities of the LR models differed among the subsamples with different sampling ratios. Although the full LR model showed that the height of a cavity is a parameter influencing roosting site use by bat groups (z = 2.36, P = 0.019), only a relatively small portion (23%, n = 100) of the models derived from the 1:1 sampling provided the same conclusion. The number of models that provide the same conclusion as the full model regarding the significance of cavity height increased as the sampling ratio grew closer to the population ratio of occupied and unoccupied cavities: 57% of models with a 1:5 sampling were significant (P < 0.05) and 83% with a 1:10 sampling (Fig 3).

Although the inference power of the LR models increased with lower presence/absence sampling ratios, their predictive power was very low. Estimates of coefficients which determined the shape of the LR curves in the subsampled models (sampling 1:1, β_0_ = -1.19±0.43, β_1_ = 0.16±0.06; sampling 1:5, β_0_ = -2.74±0.20, β_1_ = 0.15±0.03; sampling 1:10, β_0_ = -3.45±0.12, β_1_ = 0.15±0.02) differed from the parameters of the full model (β_0_ = -4.65±0.58, β_1_ = 0.16±0.06). Thus, it is clear that the probability of occurrence derived from these subsampled models will be in all cases overestimated.

The inference and the predictive power of the subsampled models were improved when we applied weights. Weighting of unoccupied cavities resulted in 65% significant models in the 1:1 sampling, 85% in the 1:5 and 94% in the 1:10 samplings. Estimates of coefficients of weighted subsampled models were also closer to the values of the full model (weighted sampling 1:1, β_0_ = -4.85±0.66, β_1_ = 0.17±0.09; weighted sampling 1:5, β_0_ = -4.78±0.21, β_1_ = 0.16±0.03; weighted sampling 1:10, β_0_ = -4.77±0.16, β_1_ = 0.16±0.02). The probability of occurrence derived from these weighted subsampled models is closer to the estimates derived from the full model (i.e. closer to reality).

## Discussion

Using the example of habitat use in a rare and hard-to-detect animal species, we have demonstrated that sampling strategy can heavily influence the reliability and predictive power of the obtained LR models. According to the full model (all dataset), the probability of Leisler’s bat presence in tree cavities increases with the cavity entrance height. This agrees with the results from other areas [9]. Such a pattern could be explained by either the thermal environment of maternity roosts, as temperature benefits arise from greater exposure of the trunks to sunlight at cavity height, and/or by an anti-predatory strategy, as higher roost entrances are safer from terrestrial predators (e.g. martens). A similar roosting pattern has also been found in other bat species (*Chalinolobus tuberculatus*: [11]; *Noctilio albiventris*: [12]; *Mystacina tuberculata*: [13]; *Barbastella barbastellus*: [14], *Nyctalus noctula*: [15, 9]; *Myotis macropus*: [16]).

Although significant use of higher roost entrances was found by the full model, most (up to 77%) of the non-weighted and/or high sampling ratio models gave a contradictory conclusion. Thus, interpretation of these models would result in the wrong ecological conclusion that the height of the cavity is insignificant with regard to roosting (i.e. a type II error). Unfortunately, 1:1 sampling is currently still the most frequently used strategy, as it occurs in more than one-third of studies published in the last 10 years (Fig 2 and S1 List). Moreover, most authors do not make any assumptions about the sampling ratio or make any attempts to correct the model by applying weights. The probability of occurrence is therefore most likely overestimated in their models. In our study, other non-weighted models with a lower ratio of occupied to unoccupied cavities (1:5 and 1:10) produced more reliable results, as the majority of them point towards the significance of entrance height. Nevertheless, the estimated values of the model coefficients which are responsible for the shape of the logistic curve strongly differed from the parameters of the full model. Such models having arbitrary sampling ratios would therefore not be usable for estimating the probability of bats’ roosting in cavities. Even if a randomly collected control sample of available cavities significantly differs from those occupied, the results would certainly overestimate the true probability value. Moreover, the risk of making a type II error is still relatively high, even in weighted models with 1:1 sampling. Adding weights caused the S-shaped curve to be lowered towards the x-axis and more closely resemble the curvature of the full model. The similarity of the estimated parameters of weighted models to the full model clearly demonstrated the importance of weights when constructing a robust LR.

The improvement of LR models demonstrated in this study was only possible due to unique information about the population ratio of occupied and unoccupied cavities. Generally, the ideal (and in most cases unreal) approach is to perform a sampling of all locations in the study area and subsequently distinguish between occupied and unoccupied locations. However, in the vast majority of cases, due to time and financial reasons, it is almost always impossible to perform such exhaustive data collection. The construction of such a full model is thus practically impossible within most practical sampling frameworks. Objective limitations force researchers to measure only a limited sample of available locations, and the resulting ratio between occupied and unoccupied locations is likely far from the true ratio, especially when an animal is rare and/or hard-to-detect in the environment. In that case we should refer to unoccupied locations as ‘available’ instead.

Some authors recommend interpreting models from case—control-studies only in terms of the odds ratio [4, 17]. Though odds ratios can be easily obtained, it could be difficult to interpret them in the context of the given problem. Model coefficients and P-values obtained from such collected data vary greatly in comparison to coefficients of a full model (Fig 3); thus, odds ratios which are calculated from the β_0_ and β_1_ would be inaccurate. Even correction of a model using weights to mimic the population ratio of occupied and unoccupied locations may not improve the model’s predictive power—mainly in the case of applying weights to a 1:1 sampling. Therefore, information about the (at least approximate) value of the population ratio is absolutely necessary.

Although Northrup et al. [17] have already examined issues regarding sampling ratios, they used only artificial data in a simulated framework and not a real-life data set, where the effects of inappropriate sampling design were explicitly demonstrated. In addition, they also recommended weighting the available sample [17], though they did not provide any practical recommendation for how to calculate the true ratio necessary for such a procedure. In contrast, we suggest that the only possible way to get at least an approximate value of the true occupied:unoccupied ratio is to apply a randomized sampling strategy. We could employ a blind map to locate occupied locations by chance in an area, and according to the number of random locations that coincide with the occupied ones, we could deduce their population ratio (e.g. transforming the study area into a grid may help to easily count occupied and unoccupied cells). Afterwards, the obtained value of the population ratio can be used to apply proper weights for unoccupied locations in the LR model (or for occupied locations, if the number of occupied locations is greater than the number of unoccupied in case of other species). Still, in the field we recommend measuring random locations at a lower ratio than 1:1 (as many as possible) and then employing weights to correct for the population ratio.

## Conclusion

Our demonstration confirmed that the most commonly employed sampling strategy representing equal numbers of occupied and unoccupied locations is the most inappropriate method in the case of hard-to-detect and/or rare species. Estimated coefficients of non-weighted models with a sampling ratio differing greatly from the population ratio were less reliable not only for inference but particularly when estimating the probability of species occurrence. Applying weights to these models strongly improved both the inference and predictive power of those models. However, to perform this procedure, it is necessary to estimate the population ratio of occupied and unoccupied locations. This estimation is possible using the randomized sampling procedure that we have herein recommended.

## Supporting information

S1 List List of 59 studies from a search using the Web of Science^™^ (All Databases) database for peer-reviewed papers published between 2005 to 2016 (combination of topic keywords ‘(logistic regression* ecology* habitat selection*) AND (telemetry OR tracking)’) which report sampling ratio between occupied and unoccupied/available locations. (DOC)

S1 Table Dataset containing GPS coordinates and heights of 932 cavities (those used by bats are marked by 1). (CSV)
