# Supplementary material for: Why sampling ratio matters: Logistic regression and studies of habitat use
Source: PLoS One. 2018 Jul 23;13(7):e0200742. doi: 10.1371/journal.pone.0200742 (PMC6056037; doi:10.1371/journal.pone.0200742)
Supplement: S1 List — (DOC) [file pone.0200742.s001.doc]

**Appendix S1.** List of 59 studies from search using the Web of Science™ (All Databases) database for peer-reviewed papers published between 2005 to 2016 (combination of topic keywords ‘(logistic regression* ecology* habitat selection*) AND (telemetry OR tracking)’) which report sampling ratio between used and unused/available locations

Aarts, G., MacKenzie, M., McConnell, B., Fedak, M. & Matthiopoulos, J. (2008) Estimating space‐use and habitat preference from wildlife telemetry data. *Ecography*, **31**, 140–160.

Alexander, S.M., Paquet, P.C., Logan, T.B. & Saher, D. J. (2005) Snow-tracking versus radiotelemetry for predicting wolf-environment relationships in the Rocky Mountains of Canada. *Wildlife Society Bulletin*, **33**, 1216–1224.

Bauder, J.M., Castellano, C., Jensen, J.B., Stevenson, D.J. & Jenkins, C.L. (2014) Comparison of movements, body weight, and habitat selection between translocated and resident gopher tortoises. *The Journal of Wildlife Management*, **78**, 1444–1455.

Beauchesne, D., Jaeger, J.A. & St-Laurent, M.H. (2013) Disentangling woodland caribou movements in response to clearcuts and roads across temporal scales. *PLoS One*, **8**, e77514.

Blanchette, P., Bourgeois, J.C. & St-Onge, S. (2007) Ruffed grouse winter habitat use in mixed softwood–hardwood forests, Québec, Canada. *Journal of Wildlife Management*, **71**, 1758–1764.

Bleich, V.C., Davis, J.H., Marshal, J.P., Torres, S.G. & Gonzales, B. J. (2009) Mining activity and habitat use by mountain sheep (*Ovis canadensis*). *European Journal of Wildlife Research*, **55**, 183–191.

Blomquist, S.M. & Hunter Jr, M.L. (2010) A multi-scale assessment of amphibian habitat selection: wood frog response to timber harvesting. *Ecoscience*, **17**, 251–264.

Briand, Y., Ouellet, J.P., Dussault, C. & St-Laurent, M.H. (2009) Fine-scale habitat selection by female forest-dwelling caribou in managed boreal forest: Empirical evidence of a seasonal shift between foraging opportunities and antipredator strategies. *Ecoscience*, **16**, 330–340.

Browne, C.L. & Paszkowski, C.A. (2014) The Influence of habitat composition, season and gender on habitat selection by western toads (*Anaxyrus boreas*). *Herpetological Conservation and Biology*, **9**, 417–427.

Copeland, J.P., Peek, J.M., Groves, C.R., Melquist, W.E., McKelvey, K.S., McDaniel, G. W., Long, C.D. & Harris, C.E. (2007) Seasonal habitat associations of the wolverine in central Idaho. *Journal of Wildlife Management*, **71**, 2201–2212.

Cruz, J., Sutherland, D.R., Anderson, D.P., Glen, A.S., Paul, J. & Leung, L.K.P. (2013) Antipredator responses of koomal (*Trichosurus vulpecula hypoleucus*) against introduced and native predators. *Behavioral Ecology and Sociobiology*, **67**, 1329–1338.

Dzialak, M.R., Olson, C.V., Harju, S.M. & Winstead, J.B. (2013). Spatial generality of predicted occurrence models of nesting habitat for the greater sage‐grouse. *Ecosphere*, **4**, 1–20.

Dzialak, M.R., Olson, C.V., Harju, S.M., Webb, S.L., Mudd, J.P., Winstead, J.B. & Hayden-Wing, L. D. (2011) Identifying and prioritizing greater sage-grouse nesting and brood-rearing habitat for conservation in human-modified landscapes. *PloS One*, **6**, e26273.

Elliot, N.B., Cushman, S.A., Macdonald, D.W. & Loveridge, A.J. (2014) The devil is in the dispersers: predictions of landscape connectivity change with demography. *Journal of Applied Ecology*, **51**, 1169–1178.

Ewald, M., Dupke, C., Heurich, M., Müller, J. & Reineking, B. (2014) LiDAR remote sensing of forest structure and GPS telemetry data provide insights on winter habitat selection of European roe deer. *Forests*, **5**, 1374–1390.

Forsman, E.D., Sovern, S.G., Taylor, M. & Biswell, B.L. (2015) Home range and habitat selection by northern spotted owls on the eastern slope of the Cascade Mountains, Washington. *Journal of Raptor Research*, **49**, 109–128.

García, P., Arévalo, V. & Lizana, M. (2010) Characterisation of den sites of American mink *Neovison vison* in central Spain. *Wildlife Biology*, **16**, 276–282.

Gastón, A., Blázquez‐Cabrera, S., Garrote, G., Mateo‐Sánchez, M.C., Beier, P., Simón, M.A. & Saura, S. (2016) Response to agriculture by a woodland species depends on cover type and behavioural state: insights from resident and dispersing Iberian lynx. *Journal of Applied Ecology*, **53**, 814–824.

Gillies, C.S., Hebblewhite, M., Nielsen, S.E., Krawchuk, M.A., Aldridge, C.L., Frair, J.L., Saher, D.J., Stevens, C.E & Jerde, C. L. (2006) Application of random effects to the study of resource selection by animals. *Journal of Animal Ecology*, **75**, 887–898.

Giroux, W., Blanchette, P., Bourgeois, J.C. & Cabana, G. (2007) Ruffed grouse brood habitat use in mixed softwood–hardwood nordic–temperate forests, Quebec, Canada. *Journal of Wildlife Management*, **71**, 87–95.

Godvik, I.M.R., Loe, L.E., Vik, J.O., Veiberg, V., Langvatn, R. & Mysterud, A. (2009) Temporal scales, trade‐offs, and functional responses in red deer habitat selection. *Ecology*, **90**, 699–710.

Gustine, D.D., Parker, K.L., Lay, R.J., Gillingham, M.P., & Heard, D.C. (2006) Interpreting resource selection at different scales for woodland caribou in winter. *Journal of Wildlife Management*, **70**, 1601–1614.

Güthlin, D., Knauer, F., Kneib, T., Küchenhoff, H., Kaczensky, P., Rauer, G., Jonozovič, M., Mustoni A. & Jerina, K. (2011) Estimating habitat suitability and potential population size for brown bears in the Eastern Alps. *Biological Conservation*, **144**, 1733–1741.

Hall, G.I., Wallace, M.C., Ballard, W.B., RUTHVEN III, D.C., Butler, M.J., Houchin, R. L., Huffman, R.T., Phillips, R.S. & Applegate, R. (2007) Rio Grande wild turkey habitat selection in the southern Great Plains. *Journal of Wildlife Management*, **71**, 2583–2591.

Hein, C.D., Castleberry, S.B. & Miller, K.V. (2008) Male Seminole bat winter roost-site selection in a managed forest. *Journal of Wildlife Management*, **72**, 1756–1764.

Holloran, M.J., Heath, B.J., Lyon, A.G., Slater, S.J., Kuipers, J.L. & Anderson, S. H. (2005) Greater sage-grouse nesting habitat selection and success in Wyoming. *Journal of Wildlife Management*, **69**, 638–649.

Hough, M.J. & Dieter, C.D. (2009) Resource selection habitat model for northern flying squirrels in the Black Hills, South Dakota. *The American Midland Naturalist*, **162**, 356–372.

Iamsiri, A. & Gale, G.A. (2008) Breeding season habitat use by Hume's pheasant *Syrmaticus humiae* in the Doi Chiang Dao Wildlife Sanctuary, Northern Thailand. *Zoological Studies*, **47**, 138–145.

Johnson, C.J. & Gillingham, M.P. (2008) Sensitivity of species-distribution models to error, bias, and model design: an application to resource selection functions for woodland caribou. *Ecological Modelling*, **213**, 143–155.

Johnson, J.B., Edwards, J.W., Ford, W.M. & Gates, J.E. (2009) Roost tree selection by northern myotis (*Myotis septentrionalis*) maternity colonies following prescribed fire in a Central Appalachian Mountains hardwood forest. *Forest Ecology and Management*, **258**, 233–242.

Joly, K. (2011) Modeling influences on winter distribution of caribou in northwestern Alaska through use of satellite telemetry. *Rangifer*, **31**, 75–85.

Klar, N., Fernández, N., Kramer-Schadt, S., Herrmann, M., Trinzen, M., Büttner, I. & Niemitz, C. (2008). Habitat selection models for European wildcat conservation. *Biological Conservation*, **141**, 308–319.

Kudo, T., Ozaki, K., Takao, G., Sakai, T., Yonekawa, H. & Ikeda, K. (2005) Landscape analysis of northern goshawk breeding home range in northern Japan. *Journal of Wildlife Management*, **69**, 1229–1239.

Lapointe, N.W.R., Thorson, J.T. & Angermeier, P.L. (2010) Seasonal meso‐and microhabitat selection by the northern snakehead (*Channa argus*) in the Potomac river system. *Ecology of Freshwater Fish*, **19**, 566–577.

LeBeau, C.W., Nielson, R.M., Hallingstad, E.C. & Young Jr, D.P. (2015) Daytime habitat selection by resident golden eagles (*Aquila chrysaetos*) in Southern Idaho, USA. *Journal of Raptor Research*, **49**, 29–42.

Leopold, C.R. & Hess, S.C. (2013) Multi-scale habitat selection of the endangered hawaiian goose: selección de hábitat a múltiples escalas de la especie en peligro branta sandvicensis. *The Condor*, **115**, 17–27.

Mancinelli, S., Peters, W., Boitani, L., Hebblewhite, M. & Cagnacci, F. (2015) Roe deer summer habitat selection at multiple spatio-temporal scales in an Alpine environment. *Hystrix, the Italian Journal of Mammalogy*, **26**, 132–140.

Mardiyanto, A., Makalew, A.D. & Higuchi, H. (2015) Spatial distribution model of stopover habitats used by oriental honey buzzards in east belitung based on satellite-tracking data. *Procedia Environmental Sciences*, **24**, 95–103.

McConville, A., Law, B.S. & Mahony, M.J. (2013) Mangroves as maternity roosts for a colony of the rare east-coast free-tailed bat (*Mormopterus norfolkensis*) in south-eastern Australia. *Wildlife Research*, **40**, 318–327.

Montalvo, A.E., Ransom Jr, D. & Lopez, R.R. (2014) Modelling greater roadrunners (*Geococcyx californianus*) habitat use in West Texas. *The Wilson Journal of Ornithology*, **126**, 359–366.

Moore, J.A. & Gillingham, J.C. (2006) Spatial ecology and multi-scale habitat selection by a threatened rattlesnake: the eastern massasauga (*Sistrurus catenatus catenatus*). *Copeia*, **2006**, 742–751.

Nelson, A.A., Kauffman, M.J., Middleton, A.D., Jimenez, M.D., McWhirter, D.E. & Gerow, K. (2016) Native prey distribution and migration mediates wolf (*Canis lupus*) predation on domestic livestock in the Greater Yellowstone Ecosystem. *Canadian Journal of Zoology*, **94**, 291–299.

Panzacchi, M., Van Moorter, B., Strand, O., Loe, L.E. & Reimers, E. (2015) Searching for the fundamental niche using individual‐based habitat selection modelling across populations. *Ecography*, **38**, 659–669.

Patthey, P., Signorell, N., Rotelli, L. & Arlettaz, R. (2012) Vegetation structural and compositional heterogeneity as a key feature in Alpine black grouse microhabitat selection: conservation management implications. *European Journal of Wildlife Research*, **58**, 59–70.

Rittenhouse, T.A. & Semlitsch, R.D. (2007) Postbreeding habitat use of wood frogs in a missouri oak-hickory forest. *Journal of Herpetology*, **41**, 645–653.

Rogala, J., Hebblewhite, M., Whittington, J., White, C., Coleshill, J. & Musiani, M. (2011) Human activity differentially redistributes large mammals in the Canadian Rockies national parks. *Ecology and Society*, **16**, 16.

Row, J.R. & Blouin-Demers, G. (2006) Thermal quality influences habitat selection at multiple spatial scales in milksnakes. *Ecoscience*, **13**, 443–450.

Ruczyński, I. & Bogdanowicz, W. (2005) Roost cavity selection by *Nyctalus noctula* and *N. leisleri* (Vespertilionidae, Chiroptera) in Białowieża Primeval Forest, eastern Poland. *Journal of Mammalogy*, **86**, 921–930.

Silvergieter, M. & Lank, D. (2011) Patch scale nest-site selection by marbled murrelets (*Brachyramphus marmoratus*). *Avian Conservation and Ecology*, **6**, 6.

Sovern, S.G., Forsman, E.D., Dugger, K.M. & Taylor, M. (2015) Roosting habitat use and selection by northern spotted owls during natal dispersal. *The Journal of Wildlife Management*, **79**, 254–262.

Stabach, J.A., Wittemyer, G., Boone, R.B., Reid, R.S. & Worden, J. S. (2016) Variation in habitat selection by white‐bearded wildebeest across different degrees of human disturbance. *Ecosphere*, **7**, e01428.

Street, G.M., Rodgers, A.R. & Fryxell, J. M. (2015) Mid‐day temperature variation influences seasonal habitat selection by moose. *The Journal of Wildlife Management*, **79**, 505–512.

Sung, Y.H., Hau, B.C. & Karraker, N.E. (2015) Spatial ecology of endangered big‐headed turtles (*Platysternon megacephalum*): Implications of its vulnerability to illegal trapping. *The Journal of Wildlife Management*, **79**, 537–543.

Tagmann-Ioset, A., Schaub, M., Reichlin, T.S., Weisshaupt, N. & Arlettaz, R. (2012) Bare ground as a crucial habitat feature for a rare terrestrially foraging farmland bird of Central Europe. *Acta Oecologica*, **39**, 25–32.

Timm, B.C., McGarigal, K. & Cook, R.P. (2014) Upland movement patterns and habitat selection of adult eastern spadefoots (*Scaphiopus holbrookii*) at Cape Cod National Seashore. *Journal of Herpetology*, **48**, 84–97.

Vonhof, M. J., & Gwilliam, J. C. (2007) Intra-and interspecific patterns of day roost selection by three species of forest-dwelling bats in Southern British Columbia. *Forest Ecology and Management*, **252**, 165–175.

Webb, S.L., Dzialak, M.R., Mudd, J.P. & Winstead, J.B. (2013) Developing spatially-explicit weighting factors to account for bias associated with missed GPS fixes in resource selection studies. *Wildlife Biology*, **19**, 257–273.

Williams, P.J., Robb, J.R., & Karns, D.R. (2012) Habitat selection by crawfish frogs (*Lithobates areolatus*) in a large mixed grassland/forest habitat. *Journal of Herpetology*, **46**, 682–688.

Zeller, K.A., McGarigal, K., Beier, P., Cushman, S.A., Vickers, T.W. & Boyce, W.M. (2014) Sensitivity of landscape resistance estimates based on point selection functions to scale and behavioral state: pumas as a case study. *Landscape Ecology*, **29**, 541–557.
